# Program Evaluation: exploring health disparities that impact chronic pain referrals within a VA Health Care System

**DOI:** 10.3389/fpain.2023.1110554

**Published:** 2023-05-09

**Authors:** Eric R. Hanson, Heidi E. Quist, Jeffrey S. Mintert, Mahreen Arshad, Brittany L. Friedman, Alexandra Pleasant, N. Stacey Monico-Cristales, Rhonda Tillman, Mark Mehelis, Anita Karnik, Anais Sonder, Aram S. Mardian

**Affiliations:** ^1^ Chronic Pain Wellness Center, Phoenix VA Health Care System, Phoenix, AZ, United States; ^2^ Department of Psychiatry, University of Arizona College of Medicine–Phoenix, Phoenix, AZ, United States; ^3^ Department of Family, Community and Preventive Medicine, University of Arizona College of Medicine–Phoenix, Phoenix, AZ, United States

**Keywords:** health disparties, chronic pain, quality improvement, equal access to care, gender disparities, age disparities, mental health disparities, race disparities

## Abstract

**Introduction:**

The present Program Evaluation study examines sociodemographic characteristics of Veterans in the Phoenix VA Health Care System who have back pain, and specifically the likelihood of those characteristics being associated with a referral to the Chronic Pain Wellness Center (CPWC) in the year 2021. We examined the following characteristics: Race/ethnicity, gender, age, mental health diagnosis, substance use disorder diagnosis, and service-connected diagnosis.

**Methods:**

Our study used cross sectional data from the Corporate Data Warehouse for 2021. 13624 records had complete data for the variables of interest. Univariate and multivariate logistic regression was used to determine the likelihood of patients' being referred to the Chronic Pain Wellness Center.

**Results:**

The multivariate model found under-referral to be significant for younger adults and for patients who identified as Hispanic/Latinx, Black/African American, or Native American/Alaskan. Those with depressive disorders and opioid use disorders, on the other hand, were found to be more likely to be referred to the pain clinic. Other sociodemographic characteristics were not found to be significant.

**Discussion:**

Study limitations include the use of cross-sectional data, which cannot determine causality, and the inclusion of patients only if the ICD-10 codes of interest were recorded for an encounter in 2021 (i.e., a prior history of a particular diagnosis was not captured). In future efforts, we plan to examine, implement, and track the impact of interventions designed to mitigate these identified disparities in access to chronic pain specialty care.

## Introduction

For almost twenty years, the Agency for Healthcare Research and Quality (AHRQ), part of the Department of Health and Human Services in the US, has been publishing National Healthcare Quality and Disparities Reports (NHQDR) to aid organizations that are working to reduce healthcare inequities. The 2021 NHQDR defines a healthcare disparity as “a difference between population groups in the way they access, experience, and receive healthcare.” (1) The factors that influence these disparities are many (1–3) and include psychosocial determinants such as race/ethnicity (4, 5), gender (6–8), age (9, 10), mental health comorbidities (11, 12) and substance use disorders (13). In pain management specifically, disparities in care seem to be influenced by similar factors.

Disparities in pain management due to race/ethnicity have long been documented. In 2012, Meghani et al. conducted a meta-analysis of research on disparities in pain treatment and reported that Black/African American individuals were the most disadvantaged racial/ethnic group and less likely than White counterparts to receive analgesic treatment for both traumatic/surgical pain and nontraumatic/nonsurgical pain. Hispanic/Latinx individuals received analgesic treatment comparable to White counterparts for traumatic/surgical pain but were less likely to receive comparable care for nontraumatic/nonsurgical pain (14). Another study found that Whites are more likely to receive analgesics than Hispanics/Latinx or Blacks/African Americans in the emergency department, that Whites receive post-op prescriptions with higher morphine milligram equivalents, and that racial/ethnic minorities receive less treatment for cancer-related pain (15). In addition to illuminating disparities in the treatment of pain, research has also reported that Blacks/African Americans experience a higher disease burden as a result of chronic pain than do Whites (16, 17). Hobson and colleagues argued that one component of this higher disease burden may be the continual stress of the social threat of racism, which, like other stressors can negatively impact pain (18).

Pain care is also influenced by gender biases. Samulowitz et al. conducted a literature review exploring gender norms and gender biases in the treatment of pain (19). In the study, healthcare professionals perceived women as more sensitive and more willing to report pain. As compared to men's reports of pain, women's were more likely to be attributed to psychological rather than somatic causes. As a result, women reporting pain received more frequent referrals to mental health care than did men. In addition, the authors found that women were prescribed fewer pain medications and fewer opioids, and women were taken less seriously by providers than men when talking about their pain (19). In contrast to the risk of dismissive pain care that women face, previous studies reflect that women can use a wider range of coping strategies than men and are more likely than men to benefit from a multimodal pain management approach (20, 21). To date, little research has been done examining disparities in pain treatment for transgender and gender diverse individuals, but available studies highlight the lack of adequate care for these patients when treated for specific pain conditions, including headaches (22) and pelvic pain (23).

Mental health and substance use disorders (SUD) also contribute to disparities in pain care. One study noted that providers who have stigmatized views of mental illness are more likely to lack confidence in patients' ability to adhere to pain treatment and subsequently offer those patients fewer pain treatment options (24). Researchers also found that mental health diagnoses influence both the patient's likelihood of reporting pain in a healthcare encounter as well as the recommended course of treatment (25). For example, patients with dementia, schizophrenia, and bipolar disorders were less likely to report being in pain, while patients with depression and PTSD were more likely to report being in pain. A similar pattern emerged for these patients when receiving pain treatment: patients with dementia, schizophrenia, and bipolar disorder were less likely to receive treatment for reported pain, whereas patients with depression and PTSD were more likely to receive pain treatment (25). In the same study, patients with SUD demonstrated higher frequency of pain reporting and were more likely to receive pain treatment (25).

While age has been acknowledged as a factor greatly impacting a patient's overall healthcare and health outcomes (26, 27), age has rarely been examined as a factor contributing to disparities in pain care. One of the few studies to explore this topic was not focused solely on age but rather included it as one of several other socioeconomic variables that impact patients' utilization of primary and tertiary care for low back pain. The researchers found that older patients used more of these resources than younger counterparts, possibly due to better insurance coverage (28). Lambert et al., studied multiple variables, including age, that were identified in systematic reviews of opioid treatment of chronic non-cancer pain. They suggested that, because of differences in pain perception and opioid metabolism, treatment of pain should be tailored differently according to patients' age (29). Finally, Reid et al. noted the high prevalence of chronic pain in older adults and recommended developing a comprehensive pain care strategy targeting this specific age group (30).

With awareness of both disparities in pain care and the significance of psychosocial determinants of health, a group of providers at the Phoenix VA Chronic Pain Wellness Center (CPWC) initiated a quality improvement project to explore potential disparities within our own system. This project examined referrals from primary care providers to the CPWC. Because of the ubiquity of back pain (31), we elected to look specifically at referrals for this common condition, and we investigated the influence of multiple psychosocial factors including race/ethnicity, gender, mental health comorbidities, substance use disorders, and a patient's service connection. We aimed to identify potential psychosocial inequities in referrals to the CPWC for back pain, and we plan to use that information to design and implement training interventions that will improve our ability to provide quality pain care to every Veteran we serve.

## Methods

### Study design and eligibility criteria

This quality improvement initiative was designed for internal purposes in support of the VA mission and was given a determination of “not research” by the Phoenix VA Research Department. Therefore, this project did not require Institutional Review Board (IRB) review and approval. The data was obtained retrospectively from the VA's Corporate Data Warehouse (CDW) by a sequel (SQL) script as part of a Program Evaluation. The data was initially screened to include only those individuals who were treated for back pain within the last year. To meet criteria, a patient had to have an encounter in the year 2021 with one of the following ICD-10 codes: M54.1, M54.16, M54.17, M54.18, M54.3, M54.30, M54.32, M54.4, M54.40, M54.41, M54.42, M54.5, M54.50, M54.51, M54.59, or M54.8. Individuals who were not treated for back pain at some point in the year 2021 were excluded from the study. 

Patients were determined to have been referred to the CPWC if a Chronic Pain Consult was placed in the year 2021, and this was coded as a dichotomous variable. In general, consults to the CPWC are placed by a primary care provider (PCP) practicing within the Phoenix VA Health Care system. Usually these consults occur at a patient's request or if their PCP feels that they would benefit from additional treatment planning, consideration of multimodal pain rehabilitation, and/or medication management for chronic pain.

The psychosocial variables of interest in this study were selected based on a literature review of factors that impact patients' access to chronic pain care. These were further limited by what archival data was available within the CDW. Psychosocial variables were defined for the analyses as follows:

#### Race/ethnicity

Six categories were available for race (Asian, Black/African American, Hawaiian/Pacific Islander, Multiracial, Native American/Alaskan, & White/Caucasian) and two for ethnicity (Hispanic/Latinx & non-Hispanic/Latinx) in patients' charts. The data was cleaned to exclude anyone who “declined to answer” race/ethnicity questions or had data that was classified as “unknown to patient.”

#### Gender

The participants' medical record gender variable only allowed for the option of male or female; however, there was an additional option for individuals to self-identify their gender on their cover sheet. Of note, only about a fourth of individuals were found to have self-identified their gender. *Male, female*, and *gender diverse* variables were all coded for this study. As the total number of gender diverse individuals in the sample was small, this group was not further divided into subcategories. Thus, our gender diverse category included the following: patients whose self-identified gender didn’t match the gender in their medical record, patients who self-identified as transgender, and patients who self-identified as “other” gender.

#### Age

Patients' age was collapsed into three categories: Patients aged 65 and older were classified as *older adults*, those 35–64 years old were classified as *middle-aged adults*, and those 18–34 years old were classified as *younger adults*.

#### Mental health diagnoses

All mental health diagnoses of interest were collapsed into five broad dichotomous categories based on ICD-10 codes and DMS-5-TR categories: *anxiety* (F41.0, F41.1, F41.3, F41.9), *depression* (F06.31, F06.32, F32.0, F32.1, F32.2, F32.3, F32.4, F32.5 F32.8, F32.89, F32.9, F33.0, F33.1, F33.2, F33.3, F33.40, F33.41, F33.42, F33.8, F33.9, F34.1), *somatization* (F54, F45.0, F45.1, F45.2*, F45.4*, F45.8, F45.9), *trauma/post-traumatic stress disorder* (PTSD; F43.0, F43.1*, F43.11, F43.2*, F43.8, F43.9), or *personality disorder* (F60.0, F60.1, F60.2, F60.3, F60.4, F60.5, F60.6, F60.7, F60.8*, F60.81, F60.89, F60.9) (32). Patients were considered to have a mental health diagnosis if any of these ICD-10 codes were recorded for a clinical encounter during the year 2021. Variables with an asterisk (*) were in the initial SQL script as possible variables of interest; however, there were no incidents of those diagnoses in the current population.

#### Substance use disorders

All substance use disorders were collapsed into five dichotomous categories, again based on ICD-10 codes: *alcohol use disorder* (AUD; any F10 code), *opioid use disorder* (OUD; any F11 code), *cannabis use disorder* (CUD; any F12 code), *tobacco use disorder* (TUD; any F17 code), and *other substance use disorder* (Other SUD; any F13, F14, F15, F16, F18, or F19 code). Patients were considered to have a substance use disorder if any of these ICD-10 codes were recorded for a clinical encounter during the year 2021.

#### Service connection

The presence of any service-connected diagnosis was treated as a dichotomous variable.

### Data analysis

After the data was recoded, SPSS version 26 was utilized for data analysis. All the data was cleaned and screened; anyone with missing or incomplete data for variables of interest was excluded from the study. Nominal variables were dummy coded for the statistical analysis using White, non-Hispanic/Latinx, younger adults, and male as the reference groups.  All logistic regression analyses were two tailed with significance level set to a *p* value <0.05. Univariate logistic regression analyses were conducted for each variable of interest in the study (age, race, ethnicity, gender, anxiety, depression, trauma/PTSD, somatization, personality disorder, AUD, OUD, CUD, TUD, Other SUD, & service connection) in order to predict the likelihood of having a consult placed to CPWC. Subsequently, a single multivariate logistic regression model combining all the aforementioned variables was utilized to reduce experiment wide error and to account for multicollinearity.

## Results

We identified 36,605 records as having at least one ICD-10 code for back pain in the year 2021. Of those records, 17,146 (46.8%) had an ICD-10 code for back pain as defined above. After cleaning and screening the data, 13,624 individuals with complete data for all variables of interest were identified and included in the analysis. The mean age of the sample was 58.32 (SD* *= ±16.72) years. Additional demographic characteristics of the sample are presented in Table 1.

**Table 1:** Psychosocial characteristics of patients seen in primary care for back pain with and without a subsequent consult to the chronic pain wellness center.

	No consult		Pain consult		Total	
Total	12,534	92.0%	1,090	8.0%	13,624	100.0%
**Age**						
>35 (young adult)*	1,364	10.0%	75	0.6%	1,439	10.6%
35–65 (middle adult)	5,900	43.3%	554	4.1%	6,454	47.4%
65+ (older adult)	5,270	38.7%	461	3.4%	5,731	42.1%
**Ethnicity**						
Non-Hispanic*	10,840	79.6%	980	7.2%	11,820	86.8%
Hispanic/Latinx	1,694	12.4%	110	0.8%	1,804	13.2%
**Race**						
White/Caucasian*	10,202	74.9%	926	6.8%	11,128	81.7%
Native American/Alaskan	216	1.6%	10	0.1%	226	1.7%
Asian	161	1.2%	13	0.1%	174	1.3%
Black/African American	1,690	12.4%	126	0.9%	1,816	13.3%
Hawaiian/Pacific Islander	132	1.0%	6	<0.1%	138	1.0%
Multiracial	133	1.0%	9	<0.1%	142	1.0%
**Gender**						
Male*	11,040	81.0%	937	6.9%	11,977	87.9%
Female	1,480	10.9%	151	1.1%	1,631	12.0%
Gender diverse	14	0.1%	2	<0.1	16	0.1%
**Mental health**						
No anxiety disorder diagnosis	10,548	77.4%	868	6.4%	11,416	83.8%
Anxiety disorder diagnosis	1,986	14.6%	222	1.6%	2,208	16.2%
No depressive disorder diagnosis	8,932	65.6%	646	4.8%	9,588	70.4%
Depressive disorder diagnosis	3,602	26.4%	434	3.2%	4,036	29.6%
No trauma/PTSD disorder diagnosis	12,271	90.1%	1,061	7.8%	13,332	97.9%
Trauma/PTSD disorder diagnosis	263	1.9%	29	0.2%	292	2.1%
No somatic symptom disorder diagnosis	12,503	91.8%	1,085	8.0%	13,588	99.7%
Somatic symptom disorder diagnosis	31	0.2%	5	<0.1%	36	0.03%
No Personality disorder diagnosis	12,390	90.9%	1,065	7.8%	13,455	98.8%
Personality disorder diagnosis	144	1.1%	25	0.2%	169	1.2%
**Substance use disorder**						
No alcohol use disorder diagnosis	11,567	84.9%	991	7.3%	12,558	92.2%
Alcohol use disorder diagnosis	967	7.1%	99	0.7%	1,066	7.8%
No Opioid use disorder diagnosis	12,240	89.8%	1,017	7.5%	13,257	97.3%
Opioid use disorder diagnosis	294	2.2%	73	0.5%	367	2.7%
No cannabis use disorder diagnosis	12,186	89.4%	1,041	7.6%	13,227	97.1%
Cannabis use disorder diagnosis	348	2.6%	49	0.4%	397	2.9%
No other substance use disorder diagnosis	12,259	90.0%	1,063	7.8%	13,322	97.8%
Other substance use disorder diagnosis	275	2.0%	27	0.2%	302	2.2%
No tobacco use disorder diagnosis	11,918	87.5%	1,020	7.5%	12,938	95.0%
Tobacco use disorder diagnosis	616	4.5%	70	0.5%	686	5.0%
**Military service connection**						
No military service connected injury	2,981	21.9%	234	1.7%	3,215	23.6%
Military service connected injury	9,553	70.1%	856	6.3%	10,409	76.4%

* Dummy coded reference variables in logistic regression.

Univariate analyses are presented in Table 2. Significant univariate analyses include: age [*R*^2^ = 0.001 (Cox & Snell), 0.003 (Nagelkerke), Model *χ*^2^(2) = 20.11, *p* < 0.001], race [*R*^2^ = 0.001 (Cox & Snell), 0.002 (Nagelkerke), Model *χ*^2^(5) = 12.67, *p* = 0.027], ethnicity [*R*^2^ = 0.001 (Cox & Snell), 0.002 (Nagelkerke), Model *χ*^2^(1) = 10.95, *p* = 0.001], anxiety disorder in 2021 [*R*^2^ = 0.001 (Cox & Snell), 0.002 (Nagelkerke), Model *χ*^2^(1) = 14.26, *p* < 0.001], depressive disorder in 2021 [*R*^2^ = 0.004 (Cox & Snell), 0.010 (Nagelkerke), Model *χ*^2^(1) = 56.25, *p* < 0.001], personality disorder in 2021 [*R*^2^ = 0.001 (Cox & Snell), 0.002 (Nagelkerke), Model *χ*^2^(1) = 8.78, *p* = 0.003], opioid use disorder in 2021 [*R*^2^ = 0.004 (Cox & Snell), 0.009 (Nagelkerke), Model *χ*^2^(1) = 53.57, *p* < 0.001] cannabis use disorder in 2021 [*R*^2^ = 0.001 (Cox & Snell), 0.002 (Nagelkerke), Model *χ*^2^(1) = 9.15, *p* = 0.002] tobacco use disorder in 2021 [*R*^2^ < 0.001 (Cox & Snell), 0.001 (Nagelkerke), Model *χ*^2^(1) = 4.43, *p* = 0.035]. Other analyses were not found to be statistically significant, including those for gender, trauma/PTSD, somatization, alcohol use disorder, other substance use disorders, and presence of a service connected injury.

**Table 2:** Univariate analyses.

	*B*	SE	Wald	(df)	*p*	Exp(*B*)
**Age**						
35–65 (middle adult)	0.54	0.13	17.85	(1)	<0.001	1.71 (1.33–2.19)
65+ (older adult)	0.46	0.13	13.13	(1)	<0.001	1.59 (1.24–2.05)
Constant	−2.90	0.12	598.16	(1)	<0.001	0.06
**Ethnicity**						
Hispanic/Latinx	−0.33	0.10	10.15	(1)	0.001	0.72 (0.59–0.88)
Constant	−2.40	0.03	5,191.66	(1)	<0.001	0.09
**Race**						
Native American/Alaskan	−0.67	0.33	4.28	(1)	0.04	0.51 (0.27–0.97)
Asian	−0.12	0.29	0.16	(1)	n.s.	0.89 (0.50–1.57)
Black/African American	−0.20	0.10	3.99	(1)	0.046	0.82 (0.68–1.00)
Hawaiian/Pacific Islander	−0.69	0.42	2.73	(1)	n.s.	0.50 (0.22–1.14)
Multiracial	−0.29	0.35	0.72	(1)	n.s.	0.75 (0.38–1.47)
Constant	−2.40	0.03	48,887.74	(1)	<0.001	0.09
**Gender**						
Female	0.18	0.09	4.01	(1)	0.045	1.20 (1.00–1.44)
Gender diverse	0.52	0.76	0.47	(1)	n.s.	1.68 (0.38–7.42)
Constant	−2.47	0.03	5,254.81	(1)	<0.001	0.09
**Mental health**						
Anxiety disorder diagnosis	0.31	0.08	15.00	(1)	<0.001	1.36 (1.16–1.59)
Constant	−2.50	0.04	5,002.50	(1)	<0.001	0.09
Depressive disorder diagnosis	0.50	0.07	58.10	(1)	<0.001	1.64 (1.44–1.87)
Constant	−2.61	0.04	4,166.93	(1)	<0.001	0.07
Trauma/PTSD disorder diagnosis	0.24	0.20	1.50	(1)	n.s.	1.28 (0.87–1.88)
Constant	−2.45	0.03	5,852.38	(1)	<0.001	0.09
Somatic symptom disorder diagnosis	0.62	0.48	1.65	(1)	n.s.	1.86 (0.72–4.79)
Constant	−2.44	0.03	5,965.25	(1)	<0.001	0.09
Personality disorder diagnosis	0.70	0.22	10.30	(1)	0.001	2.02 (1.32–3.10)
Constant	−2.45	0.03	5,905.49	(1)	<0.001	0.09
**Substance use disorder**						
Alcohol use disorder diagnosis	0.18	0.11	2.59	(1)	0.10	1.20 (0.96–1.48)
Constant	−2.46	0.03	5,511.30	(1)	<0.001	0.09
Opioid use disorder diagnosis	1.10	0.14	65.98	(1)	<0.001	2.99 (2.30–3.89)
Constant	−2.49	0.03	5,811.74	(1)	<0.001	0.08
Cannabis use disorder diagnosis	0.50	0.16	10.27	(1)	0.001	1.65 (1.21–2.24)
Constant	−2.46	0.03	5,804.41	(1)	<0.001	0.09
Other substance use disorder diagnosis	0.12	0.20	0.37	(1)	n.s.	1.13 (0.76–1.69)
Constant	−2.45	0.03	5,848.38	(1)	<0.001	0.09
Tobacco use disorder diagnosis	0.28	0.13	4.74	(1)	0.03	1.33 (1.03–1.71)
Constant	−2.46	0.03	5,677.90	(1)	<0.001	0.09
Military service connected injury	0.13	0.08	2.98	(1)	n.s.	1.14 (0.98–1.33)
Constant	−2.55	0.07	1,404.97	(1)	<0.001	0.08

The multivariate analysis was significant [*R*^2^ = 0.012 (Cox & Snell), 0.027 (Nagelkerke), Model *χ*^2^(21) = 158.46, *p* < 0.001], and results are displayed in Table 3. Older and middle-aged adults were both more likely to be referred for pain management when compared to younger adults. Hispanic/Latinx patients were less likely to be referred when compared to non-Hispanic/Latinx patients, and both Native Americans/Alaskans and Blacks/African Americans were also less likely to be referred when compared to White/Caucasian individuals. In contrast, both patients with a diagnosis of depression and those diagnosed with an opioid use disorder were more likely to be referred.

**Table 3:** Multivariate analyses.

	*B*	SE	Wald	(df)	*p*	Exp(*B*)
**Age**						
35–65 (Middle adult)	0.51	0.13	16.02	(1)	<0.001	1.67 (1.30–2.15)
65+ (Older adult)	0.51	0.13	14.89	(1)	<0.001	1.67 (1.29–2.17)
**Ethnicity**						
Hispanic/Latinx	−0.32	0.11	9.15	(1)	0.002	0.73 (0.59–0.89)
**Race**						
Native American/Alaskan	−0.68	0.33	4.33	(1)	0.04	0.51 (0.27–0.96)
Asian	−0.10	0.29	0.12	(1)	n.s.	0.90 (0.51–1.60)
Black/African American	−0.26	0.10	6.52	(1)	0.01	0.77 (0.64–0.94)
Hawaiian/Pacific Islander	−0.71	0.42	2.81	(1)	n.s.	0.49 (0.22–1.12)
Multiracial	−0.34	0.35	0.91	(1)	n.s.	0.72 (0.36–1.42)
**Gender**						
Female	0.11	0.10	1.37	(1)	n.s.	1.12 (0.93–1.35)
Gender diverse	0.31	0.09	0.16	(1)	n.s.	1.36 (0.30–6.20)
**Mental health**						
Anxiety disorder diagnosis	0.09	0.09	1.16	(1)	n.s.	1.10 (0.93–1.30)
Depressive disorder diagnosis	0.41	0.07	32.04	(1)	<0.001	1.50 (1.31–1.74)
Trauma/PTSD disorder diagnosis	0.10	0.20	0.23	(1)	n.s.	1.10 (0.74–1.63)
Somatic symptom disorder diagnosis	0.32	0.49	0.42	(1)	n.s.	1.38 (0.52–3.61)
Personality disorder diagnosis	0.43	0.23	3.54	(1)	n.s.	1.54 (0.98–2.40)
**Substance use disorder**						
Alcohol use disorder diagnosis	−0.01	0.12	0.01	(1)	n.s.	0.99 (0.78–1.25)
Opioid use disorder diagnosis	0.96	0.14	46.07	(1)	<0.001	2.61 (1.98–3.45)
Cannabis use disorder diagnosis	0.27	0.17	2.50	(1)	n.s.	1.30 (0.94–1.81)
Other substance use disorder diagnosis	−0.37	0.22	2.77	(1)	n.s.	0.69 (0.45–1.07)
Tobacco use disorder diagnosis	0.06	0.14	0.18	(1)	n.s.	1.06 (0.81–1.38)
Military service connected injury	0.12	0.08	2.21	(1)	n.s.	1.13 (0.96–1.32)
Constant	−3.14	0.15	460.45	(1)	<0.001	0.04

## Discussion

This quality improvement study presents information about back pain-related referrals to our subspecialty Chronic Pain Wellness Center (CPWC) in 2021 and also explores the relationship between those referrals and patients' psychosocial characteristics. Results reflect that 8% of patients with any back pain diagnosis (chronicity was not captured in our analysis) were referred to the CPWC, which is much higher than the 2% of chronic pain patients being managed by pain physicians as reported in a 2010 national survey (33). Of individuals referred to CPWC, about 95% identified as White/Caucasian, 11.6% identified as Black/African American, 10.1% were Hispanic/Latinx, and only 0.9% of individuals identified as Native American/Alaskan. In comparison, about 81.7% of individuals identified as white/Caucasian, 13.3% identified as black/African American, 13.2% identified as Hispanic/Latinx, and 1.7% identified as Native American/Alaskan in the total study population. Our findings that Blacks/African Americans, Hispanics/Latinx, and Native Americans/Alaskans were less likely to receive referrals are consistent with many studies that report fewer pain treatment options being available to members of marginalized ethno-racial groups (14–18). Although Hawaiian/Pacific Islander, Asian, and multiracial individuals were also referred, differences did not reach statistical significance, possibly due to smaller numbers of patients in these groups.

Our study also found that younger adults were less likely to be referred to CPWC. With the majority of the already limited research on ageism in pain treatment focused on disparities for older adults (34–36), it is notable that our project found a disparity for younger patients. The reason for our finding is unclear. One possible explanation is the VA Stepped Care Model for Pain Management, which encourages lower complexity patients with chronic pain to be managed within primary care rather than specialty care (37, 38). Thus, as younger patients may have fewer comorbidities and may have trialed fewer treatments, primary care providers may manage a larger proportion of younger patients with chronic pain within primary care. However, given what we know about neuroplasticity and the increasing complexity of pain over time (39), earlier referral is likely to be more helpful for long-term management of chronic pain and may also prevent patients from overutilizing biomedical treatments for a condition that is better served by a whole person approach (40, 41).

Patients with depression were found to be more likely to be referred to the CPWC, as were patients with opioid use disorder. About 39.8% of individuals referred had a diagnosis of depressive disorder compared to 29.6% of the total study population, and about 6.7% had a diagnosis of opioid use disorder compared to 2.7% of the total study population. These findings partially mirror results of one study that reported increased likelihood of receiving treatment for reported pain in patients with depression, substance use disorders, and PTSD (25). However, a trauma/PTSD diagnosis, was not found to have a significant influence on referrals in our study. Additionally, opioid use disorder was the only substance use disorder we found to be significant. Indeed, in the current study population, 0.02% (*n* = 292) of participants were identified as having a trauma/PTSD diagnosis compared to an estimated 11%–20% prevalence of PTSD diagnoses documented for the OEF/OIF Veteran cohort, 12% for the Gulf War/Desert Storm era, and 15% for the Vietnam Veteran population (42). While our study methods may have underrepresented rates of all of the mental health diagnoses examined because we only included patients with a mental health diagnosis coded in an encounter in 2021 (i.e., a mental health diagnosis on a problem list was not included), it is possible that PTSD specifically was especially underrepresented. PCPs frequently treat depression at the Phoenix VA, but PTSD is often referred to a mental health provider for diagnosis and treatment. Therefore, a diagnosis of PTSD might be less likely than a diagnosis of depression to be coded in an encounter with a PCP.

Higher referral rates specifically for individuals with opioid use disorder may be the result of the expertise of CPWC medical providers in our pain clinic, all of whom are buprenorphine waivered and regularly evaluate and treat opioid use disorder/opioid dependence. A significant portion of our medical providers are also board certified in addiction medicine, and furthermore, the CPWC provides an integral educational experience for University of Arizona College of Medicine—Phoenix Addiction Medicine Fellowship Program.

Gender was not found to be a statistically significant factor in CPWC referrals, which appearsinconsistent with a long history of literature detailing disparities in pain care for females (19, 21), but is consistent with the findings of a recent national comprehensive questionnaire given to male and female post-9/11 Veterans between 2016 and 2019, which found that about the same percentage of females had received recent treatment for severe chronic pain as males (38.3% compared to 37.1%, respectively) (43). However, a 2022 phenomenological study of 13 military women with chronic pain found that, despite the VA's recent efforts to optimize and standardize the treatment of pain, female Veterans continue to report unconscious gender bias when discussing their pain with providers (44). Therefore, even though female patients may be offered equal access to pain care, there is still work to be done to improve our female Veterans' pain care experience. It is also important to continue to evaluate and improve access to pain care for patients who are transgender and gender diverse. Our finding that being gender diverse did not impact referrals to CPWC contrasts with emerging literature that illuminates disparities in healthcare for patients who do not identify as cisgender (45). Our findings for gender diverse patients likely did not reach significance because the study was underpowered for this category (*n* = 16).

Like gender, the presence or absence of a service-connected diagnosis was not found to be statistically significant. Veterans may apply for service connection if they became sick or injured during their military service or if military service worsened an existing condition (46). Service connection was evaluated in our project because of evidence that interacting with a disability compensation system is a strong predictor of pain-related disability (47). Our failure to find significance does not rule out a connection between Veterans' service connection and referral to pain management. The results may have been different if we had been able to look only at pain-related service connections. As it was, service connections ranging from tinnitus to prostate cancer to lumbar strain were synonymous in the analysis.

There were cases in which the univariate and multivariate logistic regression analyses varied in their significance. For example, the univariate logistic regression analysis for tobacco use disorder was significant; however, when this variable was placed in the multivariate analysis, it was not a significant predictor. An explanation for this finding might be shared variance within the univariate variables and the multivariate analysis' reduced multicollinearity.

## Limitations

Our quality improvement project has several limitations. The present study exclusively utilized cross-sectional data from 2021 that was pulled in 2022. Because of our reliance on ICD-10 codes, we may have miscategorized some patients due to inherent coding inaccuracies and inconsistencies (48). Patients may also have been miscategorized as a result of the timing of their healthcare encounters. For example, a patient who had a primary care encounter with an ICD-10 code for back pain in late 2021 may have been incorrectly labeled as not being referred to the CPWC if their primary care provider did not place the consult to CPWC until early 2022. Additionally, we did not examine prior instances of ICD-10 codes or codes on patient problem lists. Therefore, a patient diagnosed with depression prior to 2021, whose provider entered an ICD-10 code for back pain in 2021 but did not enter one for depression, would not be classified as a patient with depression for the analysis. The benefit of this strategy was an increased likelihood of the patient's mental health disorder being active at the time of the consult.

Another limitation of our study is the lack of nuanced information about why referrals were or were not placed. Providers' personal thresholds for placing a pain management consult, patient-provider dynamics during each appointment, and providers' implicit biases are not captured by our analyses in this context. Additionally, we operated under the assumption that the majority of the data was from patients with chronic lower back pain; it is possible that some acute or subacute back pain encounters were also included within our analysis. Unfortunately, there was no way for us to determine acute/subacute vs. chronic pain from the data available to us within the CDW. Speculation on how these factors might impact the referral process is beyond the current study.

Finally, our study was conducted during the COVID-19 pandemic and in the year after many patients and providers experienced shelter-in-place orders that took a toll on physical and mental health. In 2021, we also saw the continuation of virtual healthcare delivery for many patients, which may have influenced referral patterns to specialty services such as CPWC. The pandemic may also have influenced systemic and interpersonally marginalizing interactions. The impact of COVID-19 on our project results is unclear. As we move forward with future iterations of this project, we may be able to gain more insight into what, if any, effects the pandemic had on referrals to CPWC.

## Future directions

The current quality improvement project highlights several disparities in referrals to the CPWC based on various sociodemographic variables. However, it is not possible to conclude what factors might contribute to these disparities. As such, future research is needed to identify the mechanisms underlying the disparities. For example, it may be useful to explore other potentially moderating variables such as patients' socioeconomic status, service-connection rating, disability status, employment status, or education level. The amount of time a PCP spends with a patient or providers' educational background and demographics may also be found to have an impact on referral disparities. Future researchers might also consider assessing providers' self-reported competence in treating chronic pain or patients' self-reported experiences with marginalization in medical settings.

As we continue to look for other factors that may be influencing referral disparities, we also hope to design and implement interventions that will begin to reduce disparities. We will explore methods of disseminating information to PCPs about populations that were underserved in this study and will also consider offering evidence-informed and thoughtfully designed training on reducing implicit bias (49–51). Other strategies may include standardizing the referral process and implementing continuing education for primary care staff regarding who would benefit from a CPWC referral. Lastly, the CPWC team intends to continue periodic assessments of the psychosocial characteristics of Veterans referred to our clinic with the aim of determining whether our mitigation strategies are effective.

## Conclusion

In this quality improvement project, we examined referrals to our Chronic Pain Wellness Center for back pain in the year 2021. We found that patients with depression and OUD were more likely whereas patients who were younger, Hispanic/Latinx, Black/African American, or Native American/Alaskan were less likely to be referred to our clinic. Our next step will be to design and implement an intervention that targets these disparities to improve pain care access for all Veterans who would benefit from it.

## Data Availability

The dataset was obtained from the VA's Corporate Date Warehouse (CDW). Questions regarding the dataset should be directed to VHAPHOFOIA@va.gov.
