# Supplementary material for: Program Evaluation: exploring health disparities that impact chronic pain referrals within a VA Health Care System
Source: Front Pain Res (Lausanne). 2023 May 9;4:1110554. doi: 10.3389/fpain.2023.1110554 (PMC10204586; doi:10.3389/fpain.2023.1110554)
Supplement: Supplementary file 1 [file Table1.docx]

**Table 1: Psychosocial characteristics of patients seen in primary care for back pain with and without a subsequent consult to the Chronic Pain Wellness Center**

| Variable | No Consult | | Pain Consult | | Total | |
| --- | --- | --- | --- | --- | --- | --- |
| Total | 12534 | 92.0% | 1090 | 8.0% | 13624 | 100.0% |
| Age |  |  |  |  |  |  |
| >35 (Young Adult) * | 1364 | 10.0% | 75 | 0.6% | 1439 | 10.6% |
| 35-65 (Middle Adult) | 5900 | 43.3% | 554 | 4.1% | 6454 | 47.4% |
| 65+ (Older Adult) | 5270 | 38.7% | 461 | 3.4% | 5731 | 42.1% |
| Ethnicity |  |  |  |  |  |  |
| Non-Hispanic * | 10840 | 79.6% | 980 | 7.2% | 11820 | 86.8% |
| Hispanic/Latinx | 1694 | 12.4% | 110 | 0.8% | 1804 | 13.2% |
| Race |  |  |  |  |  |  |
| White/Caucasian * | 10202 | 74.9% | 926 | 6.8% | 11128 | 81.7% |
| Native American / Alaskan | 216 | 1.6% | 10 | 0.1% | 226 | 1.7% |
| Asian | 161 | 1.2% | 13 | 0.1% | 174 | 1.3% |
| Black / African American | 1690 | 12.4% | 126 | 0.9% | 1816 | 13.3% |
| Hawaiian / Pacific Islander | 132 | 1.0% | 6 | <0.1% | 138 | 1.0% |
| Multiracial | 133 | 1.0% - | 9 | <0.1% | 142 | 1.0% |
| Gender |  |  |  |  |  |  |
| Male * | 11040 | 81.0% | 937 | 6.9% | 11977 | 87.9% |
| Female | 1480 | 10.9% | 151 | 1.1% | 1631 | 12.0% |
| Gender Diverse | 14 | 0.1% | 2 | <0.1 | 16 | 0.1% |
| Mental Health |  |  |  |  |  |  |
| No Anxiety Disorder Diagnosis | 10548 | 77.4% | 868 | 6.4% | 11416 | 83.8% |
| Anxiety Disorder Diagnosis | 1986 | 14.6% | 222 | 1.6% | 2208 | 16.2% |
| No Depressive Disorder Diagnosis | 8932 | 65.6% | 646 | 4.8% | 9588 | 70.4% |
| Depressive Disorder Diagnosis | 3602 | 26.4% | 434 | 3.2% | 4036 | 29.6% |
| No Trauma/PTSD Disorder Diagnosis | 12271 | 90.1% | 1061 | 7.8% | 13332 | 97.9% |
| Trauma/PTSD Disorder Diagnosis | 263 | 1.9% | 29 | 0.2% | 292 | 2.1% |
| No Somatic Symptom Disorder Diagnosis | 12503 | 91.8% | 1085 | 8.0% | 13588 | 99.7% |
| Somatic Symptom Disorder Diagnosis | 31 | 0.2% | 5 | <0.1% | 36 | 0.03% |
| No Personality Disorder Diagnosis | 12390 | 90.9% | 1065 | 7.8% | 13455 | 98.8% |
| Personality Disorder Diagnosis | 144 | 1.1% | 25 | 0.2% | 169 | 1.2% |
| Substance Use Disorder |  |  |  |  |  |  |
| No Alcohol Use Disorder Diagnosis | 11567 | 84.9% | 991 | 7.3% | 12558 | 92.2% |
| Alcohol Use Disorder Diagnosis | 967 | 7.1% | 99 | 0.7% | 1066 | 7.8% |
| No Opioid Use Disorder Diagnosis | 12240 | 89.8% | 1017 | 7.5% | 13257 | 97.3% |
| Opioid Use Disorder Diagnosis | 294 | 2.2% | 73 | 0.5% | 367 | 2.7% |
| No Cannabis Use Disorder Diagnosis | 12186 | 89.4% | 1041 | 7.6% | 13227 | 97.1% |
| Cannabis Use Disorder Diagnosis | 348 | 2.6% | 49 | 0.4% | 397 | 2.9% |
| No Other Substance Use Disorder Diagnosis | 12259 | 90.0% | 1063 | 7.8% | 13322 | 97.8% |
| Other Substance Use Disorder Diagnosis | 275 | 2.0% | 27 | 0.2% | 302 | 2.2% |
| No Tobacco Use Disorder Diagnosis | 11918 | 87.5% | 1020 | 7.5% | 12938 | 95.0% |
| Tobacco Use Disorder Diagnosis | 616 | 4.5% | 70 | 0.5% | 686 | 5.0% |
| Military Service Connection |  |  |  |  |  |  |
| No Military Service Connected Injury | 2981 | 21.9% | 234 | 1.7% | 3215 | 23.6% |
| Military Service Connected Injury | 9553 | 70.1% | 856 | 6.3% | 10409 | 76.4% |

 * Dummy coded reference variables in logistic regression
